# Bridging the gap between AI and traditional teaching methods in medical education: opportunities, challenges, and solutions

**DOI:** 10.3389/fmed.2026.1820545

**Published:** 2026-05-11

**Authors:** Yuanting Li, Wei Wang, Yan Zhang, Suxiang Li, Xue Mei, Bo Huang

**Affiliations:** 1 School of Clinical Medicine and Affiliated Hospital, North Sichuan Medical College, Nanchong, China; 2 Department of Finance and Accounting, North Sichuan Medical College, Nanchong, China; 3 Department of Intensive Care Unit, Nanchong Traditional Chinese Medicine Hospital, Nanchong, China; 4 College of Pharmacy, North Sichuan Medical College, Nanchong, China

**Keywords:** artificial intelligence, clinically validated AI, ethical challenges, ethical governance framework, generative AI, humanistic care, humanistic values, medical education

## Abstract

The integration of artificial intelligence (AI) into medical education has given rise to the innovative “AI + X” model, in which X refers to core disciplines such as clinical medicine, pharmacy, and nursing. Although this model enables personalized learning and immersive practical training, its widespread implementation also raises urgent ethical challenges. This study identifies key risks in AI-enabled medical education, including algorithmic bias that may shape students’ professional perceptions and data privacy breaches resulting from unauthorized data use. A critical contribution of this study is the clear differentiation between generative AI tools (e.g., ChatGPT) and clinically validated AI systems (e.g., FDA-cleared radiology diagnostic tools), a distinction often overlooked in current curricula. The paper further examines the risks of using AI as a decision-support or moral guide, which may weaken humanistic values and professional autonomy, and discusses how superficial AI use may misalign with educators’ roles, impair the patient-physician relationship, and foster student over-reliance. To address these challenges, we propose strengthening oversight of data processing and storage, improving transparency in AI decision-making, preserving human agency, fostering emotional engagement, enhancing educators’ integrated teaching competencies, and cultivating students’ critical thinking. Ultimately, the responsible integration of AI into medical education requires collaborative action from all stakeholders and should be guided by a three-dimensional ethical governance framework encompassing technological ethics, educational philosophy, and medical humanities.

## 1 Background

The rapid advancement of science and technology worldwide, accompanied by the emergence of large language models (LLMs), has ushered in the era of artificial intelligence (AI). This transformative period has given rise to the “AI + X” model, in which X represents the core disciplinary domains of medical education (e.g., clinical practice, pharmaceutical science, and critical care medicine), and has reshaped traditional teaching and learning methods through the integration of AI technologies into specialized medical training (1).

In medical education, this paradigm is driving innovations that enhance both the quality and accessibility of training, making it more personalized and adaptive to students’ needs and the evolving demands of the healthcare sector (1–3). AI applications in medical education can be broadly divided into two categories that require explicit differentiation in curricular design: generative AI tools and clinically validated AI systems (4, 5). Generative AI tools (e.g., ChatGPT and Bard) can generate human-like text and clinical reasoning frameworks, but they lack regulatory approval and standardized diagnostic performance, and their outputs may contain plausible yet inaccurate medical information (6, 7). In contrast, clinically validated AI systems (e.g., FDA-cleared diabetic retinopathy screening systems such as IDx-DR, or task-specific radiology AI systems that have undergone external validation and regulatory review) undergo rigorous prospective and retrospective validation and demonstrate measurable sensitivity, specificity, and positive and negative predictive values for defined clinical tasks (4, 8). These validated systems are integrated into clinical workflows to augment physician decision-making, whereas generative AI currently functions primarily as a supplementary learning tool for medical students.

Accordingly, an important objective of AI-related medical curricula is to help students distinguish between generative AI as a language-based assistive tool for learning and reflection, and clinically validated AI as a task-specific decision-support technology with measurable diagnostic performance and regulatory oversight.

At the same time, AI applications, including virtual reality (VR) surgical training simulations, adaptive learning systems for personalized curriculum pacing, and clinically validated medical image analysis tools, have significantly enriched medical education by providing immersive and hands-on learning experiences (1, 9).

These tools allow medical students to engage in realistic and interactive scenarios, practice complex procedures, and improve clinical decision-making in relatively low-risk environments. AI-driven platforms can also support personalized learning pathways by adjusting content and pacing according to individual progress, thereby addressing diverse learning needs among students.

In parallel, the growing demand for highly skilled medical professionals has stimulated the development of innovative educational models, with AI playing an increasingly important role in shaping the next generation of healthcare providers. The rise of “AI + medical” interdisciplinary expertise has also created new career opportunities across both the medical and technology sectors. As healthcare systems worldwide increasingly rely on AI to improve diagnostic accuracy, treatment outcomes, and operational efficiency, the integration of AI into medical education has become both timely and necessary (9).

However, alongside these advances, significant ethical challenges must be addressed to ensure that AI integration in medical education is both responsible and beneficial. Algorithmic bias, for example, can perpetuate stereotypes and contribute to discriminatory practices in medical training, potentially affecting students’ career trajectories and healthcare delivery (10). Privacy breaches and concerns regarding the security of sensitive patient data further complicate the use of AI, especially when large datasets are required for model development and deployment (11).

In addition, the potential erosion of humanistic care remains a serious concern. This concern, however, should not frame technological literacy and humanistic values as a binary opposition. Rather, AI-related technical competence, including the ability to evaluate the limitations of AI systems, can itself be understood as an expression of humanistic care, because it helps protect patients from harm caused by over-reliance on unvalidated or biased AI tools (12).

**Figure 1:**
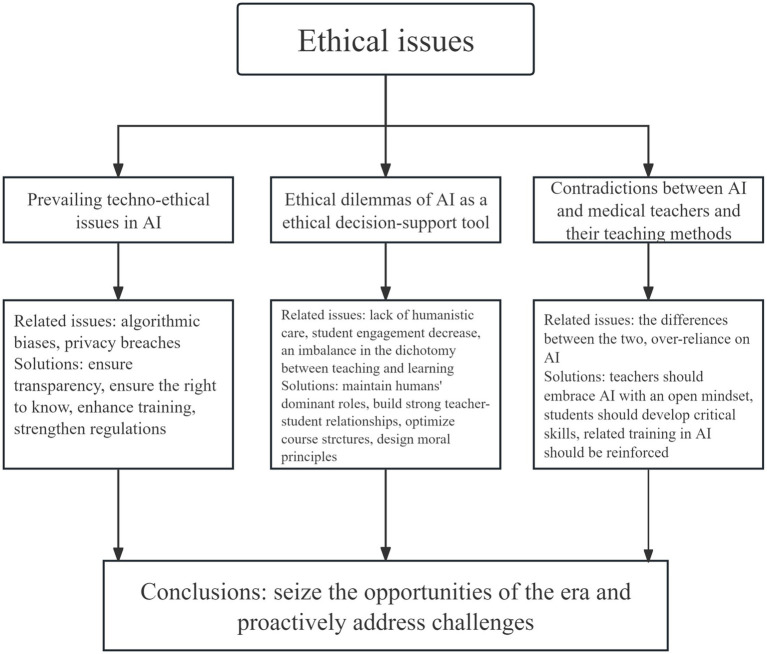
Ethical issues and solutions in the integration of AI into medical education.

These ethical dilemmas are summarized in Figure 1, which outlines the principal challenges and corresponding responses related to the integration of AI into medical education. As the healthcare sector continues to incorporate AI into training and practice, it is essential to ensure that technological advances enhance, rather than replace, the humanistic foundations of medicine. This requires collaboration among educators, AI developers, and policymakers to establish appropriate ethical guidance and safeguard the future of medical education in an increasingly AI-driven world.

## 2 Prevalent techno-ethical issues in AI integration for medical education

### 2.1 Core issues: algorithmic bias and data privacy breaches

Algorithmic bias in LLMs refers to systematic inaccuracies or stereotypical patterns that reflect the social and cultural biases embedded in their training data. These biases manifest differently in generative AI and clinically validated AI systems. In generative AI (e.g., ChatGPT), bias may appear in the form of gendered or racialized clinical recommendations, such as downplaying cardiovascular risk in female patients (10). In clinically validated AI systems, bias more often results from imbalanced training datasets, for example, the underrepresentation of non-Caucasian populations in dermatology AI training, which may reduce the accuracy of skin cancer detection in minority groups (10, 13).

Such biases may have harmful consequences, including the reinforcement of stereotypes, the spread of misinformation, and the formation of misleading clinical assumptions (10, 14). When medical educators or students rely on such distorted outputs, existing prejudices may be reinforced, and inaccurate medical concepts may be carried into future clinical practice (8). For example, gendered patterns in ChatGPT outputs may influence how some students perceive and respond to patients during clinical training (10). Similarly, one illustrative example of dataset bias in clinical AI is the underrepresentation of low-income and rural populations in chronic disease management models, which may contribute to misclassification of disease severity and suboptimal treatment recommendations in these groups (13).

Data privacy is another major concern in AI-enabled medical education. The risk may be greater with generative AI tools, which often store user inputs on third-party servers without explicit medical data protection protocols, in contrast to clinically validated AI systems that are generally subject to formal requirements such as HIPAA (Health Insurance Portability and Accountability Act) and GDPR (General Data Protection Regulation) for data storage and processing (11). Additionally, the lack of robust legal and ethical frameworks governing AI-related educational research remains problematic, partly because of the concentration of research activity in technologically advanced regions and the dominance of privately owned platforms and applications, which may not always provide sufficient transparency or regulatory accountability (9). These issues have raised substantial concerns among medical educators regarding data privacy, storage security, and the interpretability of AI systems used in educational contexts.

### 2.2 Targeted solutions for techno-ethical issues

To address algorithmic bias, medical schools and educators should equip students with the technical competencies needed to evaluate AI system performance, moving beyond abstract calls for “transparency” alone (13). Students should be taught to recognize the “accuracy paradox,” in which high overall accuracy may mask poor performance in minority subgroups; identify training shortcuts, such as reliance on demographic proxies rather than clinically meaningful features; and distinguish between internal validation (testing on the same dataset used for training) and external validation (testing on independent and diverse patient cohorts), a key step in assessing real-world AI performance (13, 15). Internal validation may demonstrate technical consistency, but only external validation across independent institutions and diverse patient populations can support confidence in generalizability for clinical education and practice. For example, students should understand that a model showing 92% overall accuracy may still perform poorly in underrepresented patient subgroups, making “high accuracy” an incomplete indicator of safety in clinical use. Medical schools and educators should also promote transparency and interpretability in AI-supported decision-making processes (16–18), as both students and patients have a legitimate interest in understanding how such systems generate their outputs. At the institutional level, AI developers and medical organizations should strengthen internal policies and procedures for data processing and storage in order to reduce privacy risks (19). Where generative AI tools are used in medical education, they should, where feasible, be configured to comply with medical data protection requirements, for example, by de-identifying patient and student information and using local rather than cloud-based data storage (8). Clinically validated AI systems, by contrast, should maintain ongoing compliance with applicable regulatory standards for data security (4). Privacy-preserving approaches such as federated learning and homomorphic encryption may help balance data utility and privacy protection by enabling model training without direct exposure of raw data (20). In addition, ethical guidelines and assessment tools are needed to support the morally responsible use of AI in medical education. During implementation, both external oversight of data and algorithms and internal monitoring of AI-supported programs may help ensure that AI applications remain within acceptable ethical and regulatory boundaries (19). It is also important to strengthen the ethical awareness of AI-related technical personnel, encourage socially responsible innovation, and promote closer alignment between technological development and human values, thereby helping to prevent ethical problems at their source (21).

## 3 Ethical dilemmas of AI as a moral judge or ethical decision-support tool

### 3.1 Core issues: erosion of humanistic values and reduced student engagement

Although advancements in science and technology are essential for medical progress, the development of medical humanities has often lagged behind (22). The integration of AI into medical humanities education may further widen this gap if not implemented with intentional pedagogical design (5, 14, 22–24). A key concern is that AI, particularly generative AI, may prioritize technological efficiency over humanistic care, potentially contributing to the erosion of humanistic values in medicine (19). In addition, the “goodness” associated with medical humanities may be reduced to probabilistic representations due to the algorithmic nature of AI, which may not align well with the complex, context-dependent ethical dilemmas encountered in clinical practice (e.g., end-of-life decision-making and resource allocation in critical care) (19). As a result, the depth and relational aspects of medical humanities may be insufficiently conveyed, potentially weakening the central role of human judgment. As AI technologies continue to evolve, they may increasingly function as decision-support tools, which could contribute to student over-reliance on AI and reduced attention to empathy and compassion in clinical practice.

Student engagement and motivation may also be affected by superficial forms of AI integration (24). Simply replacing traditional in-person teaching with AI-supported or online collaborative learning may not adequately sustain engagement, particularly when such platforms lack the emotional and social interactions that are central to medical humanities education (24). AI may also disrupt the balance between teaching and learning in medical education, making it overly reliant on algorithms and neglecting the human connections that are essential to the field (19). In this context, AI may disrupt the balance between teaching and learning by overemphasizing algorithmic processes while underrepresenting the human relationships that are fundamental to medical practice. Over time, this imbalance may diminish the affective and relational dimensions of medical education.

### 3.2 Solutions: safeguard human dominance and integrate emotional engagement

When applying AI in medical humanities education, it is important to recognize its potential limitations and to implement measures that preserve both educational quality and humanistic values (25). A central principle is to maintain human agency in the integration of AI and medical humanities. Building on this principle, a legally and ethically grounded AI ecosystem may help ensure that AI supports, rather than displaces, the development of humanistic competencies while addressing emerging ethical challenges (26). For example, AI may be used to curate clinical cases involving ethical dilemmas (e.g., pediatric informed consent or medical futility), but the interpretation and discussion of these cases should be guided by human educators to support the application of humanistic reasoning (5).

In distance or hybrid learning environments, fostering emotional connections among students, educators, and peers remains essential for effective learning outcomes (24). When transitioning from traditional teaching to AI-supported formats, course design should be carefully adapted, for instance, by combining AI-driven virtual patient simulations with facilitated small-group discussions that explicitly address the humanistic dimensions of patient care (27). Given the rapid expansion of digital technologies, further research is needed on how digital learning environments can effectively support medical humanities education, alongside the development of curricula that explicitly address ethical principles and humanistic values (28). Importantly, such curricula should avoid framing technological competence and humanistic care as opposing domains. Instead, they should emphasize that the ability to critically evaluate AI systems, including the identification of bias, can itself contribute to patient-centered care and the promotion of equity in clinical practice (12).

## 4 Contradictions between AI and medical educators’ traditional teaching methods

### 4.1 Core issues: pedagogical misalignment and student over-reliance on AI

Although AI-enhanced education can improve efficiency and support standardized skill acquisition (9), its pedagogical core does not fully align with that of traditional medical education. This structural misalignment is reflected primarily in two respects: the tendency of AI-enhanced education to prioritize efficiency over moral formation, and its emphasis on standardized learning pathways over the context-dependent clinical reasoning cultivated through traditional medical training (Table 1).

**Table 1:** Differences between AI-enhanced education and traditional medical education and their educational implications.

Comparative aspects	AI-enhanced education	Traditional education	Educational implications
Pedagogical core	Efficiency first; data-driven skill acquisition	Moral education first; promote moral integrity and nurture people	AI’s focus on efficiency may lead students to prioritize clinical outcomes over humanistic care (e.g., rapid diagnosis over patient communication) without intentional curricular intervention; traditional education’s strength in moral modeling must be preserved and integrated with AI training.
Main focus	Transfer of knowledge and skills; standardized learning pathways	Interactive, social and generative nature of education; context-dependent clinical reasoning	AI excels at delivering foundational knowledge and technical skill training but lacks the ability to teach nuanced, context-dependent clinical reasoning (a core competency developed via traditional apprenticeship); traditional education’s interactive elements (e.g., bedside teaching) must complement AI’s standardized training.

This pedagogical misalignment may have two major consequences for medical education. First, students may prioritize efficiency over humanistic care if AI-enhanced education is not balanced by traditional moral and interpersonal teaching (19). This can potentially exacerbate tensions in the doctor-patient relationship and negatively impact the long-term development of the healthcare sector. For example, students trained primarily via AI-powered diagnostic tools may lack the communication skills to explain a diagnosis to a patient or address their emotional concerns. This may exacerbate tensions in the doctor-patient relationship and affect the long-term development of the healthcare sector. For example, students trained primarily with AI-supported diagnostic tools may have limited opportunities to develop the communication skills needed to explain diagnoses and respond to patients’ emotional concerns (29). Second, the use of AI, particularly generative AI, may contribute to student over-reliance when chatbots are used to generate clinical notes or answer clinical reasoning questions without sufficient critical evaluation (6). Over-reliance of this kind may weaken independent clinical judgment, such as the ability to interpret medical images without assistance, and may ultimately affect patient care when students enter clinical practice (29, 30). For instance, uncritical use of ChatGPT to draft discharge summaries could introduce errors in medication instructions or follow-up recommendations if the generated content is not carefully verified (6).

### 4.2 Practical solutions for pedagogical integration

#### 4.2.1 Empower medical educators with AI literacy and integrated teaching skills

Medical educators should engage with the digital era openly and develop a clear understanding of the differences between generative AI and clinically validated AI, as well as their respective capabilities and limitations (4, 5). They should also reflect critically on AI applications and remain attentive to their ethical implications and educational values (19). Educators can strengthen their ability to integrate AI into teaching by developing familiarity with the principles and applications of AI and incorporating appropriate tools into teaching practice (31). For example, clinically validated AI image-analysis tools may be used in bedside teaching to compare AI-assisted and human diagnostic interpretations, while generative AI may be used to develop diverse clinical case scenarios for small-group discussion (27). In some contexts, educators may also adapt AI tools to better align with educational goals, for example by limiting unvalidated clinical recommendations, which requires continuing professional development in both conceptual understanding and practical skills (31). A possible faculty development model is an AI in Medical Education workshop series that includes modules on AI classification, AI performance evaluation, and integrated AI lesson-plan design (32, 33).

#### 4.2.2 Cultivate students’ critical thinking and responsible AI use

Medical students should learn to use AI systems effectively through supervised hands-on experience, including guided use of AI tools and participation in case-based discussion (19). They also need to develop the critical thinking skills required to assess the advantages, limitations, and risks of AI tools, including training in machine-learning performance metrics (e.g., sensitivity, specificity, positive predictive value, and negative predictive value) and AI bias evaluation (13, 15). For example, medical schools may design a laboratory-based exercise in which students test a dermatology AI model on diverse patient datasets and analyze subgroup performance across racial and ethnic groups (10, 13). A core curricular competency is students’ ability to classify an AI tool before using it: that is, to determine whether it is a generative model, a predictive algorithm, or a clinically validated decision-support system, and to judge the appropriate level of trust, supervision, and clinical applicability for each.

A practical curricular module could include four components: (1) AI tool classification (generative vs. predictive vs. clinically validated systems); (2) interpretation of performance metrics (sensitivity, specificity, PPV, NPV, calibration, subgroup performance); (3) validation appraisal (internal vs. external validation, dataset shift, generalizability); and (4) bias detection exercises using real or simulated clinical datasets. Students’ competence may then be assessed through case-based critique assignments rather than passive knowledge recall.

Medical students should also continue to value the uniquely human dimensions of medical practice, such as empathy and communication, so that AI complements rather than displaces the physician’s role in healthcare (29). One possible curricular model is an AI + Humanistic Care clinical rotation in which students use AI diagnostic tools alongside traditional patient interviews and are evaluated on both diagnostic accuracy and patient communication (32, 34).

#### 4.2.3 Strengthen ethical and legal oversight of AI in medical education

The ethical and legal dimensions of AI use in medical education should be strengthened to help ensure that students use these technologies safely and appropriately (19). Medical schools should develop clear institutional policies that distinguish between acceptable and unacceptable uses of generative AI, for example, allowing AI to assist with drafting note outlines while prohibiting unreviewed AI-generated final clinical notes (6). Technical personnel should also support the development of teaching resources and implementation strategies that help keep AI-enhanced education interactive, socially grounded, and educationally meaningful. Above all, the moral and humanistic dimensions of medical practice should remain central so that efficiency does not become the sole guiding value of medical education (19).

## 5 The three-dimensional ethical governance framework for AI in medical education

**Figure 2:**
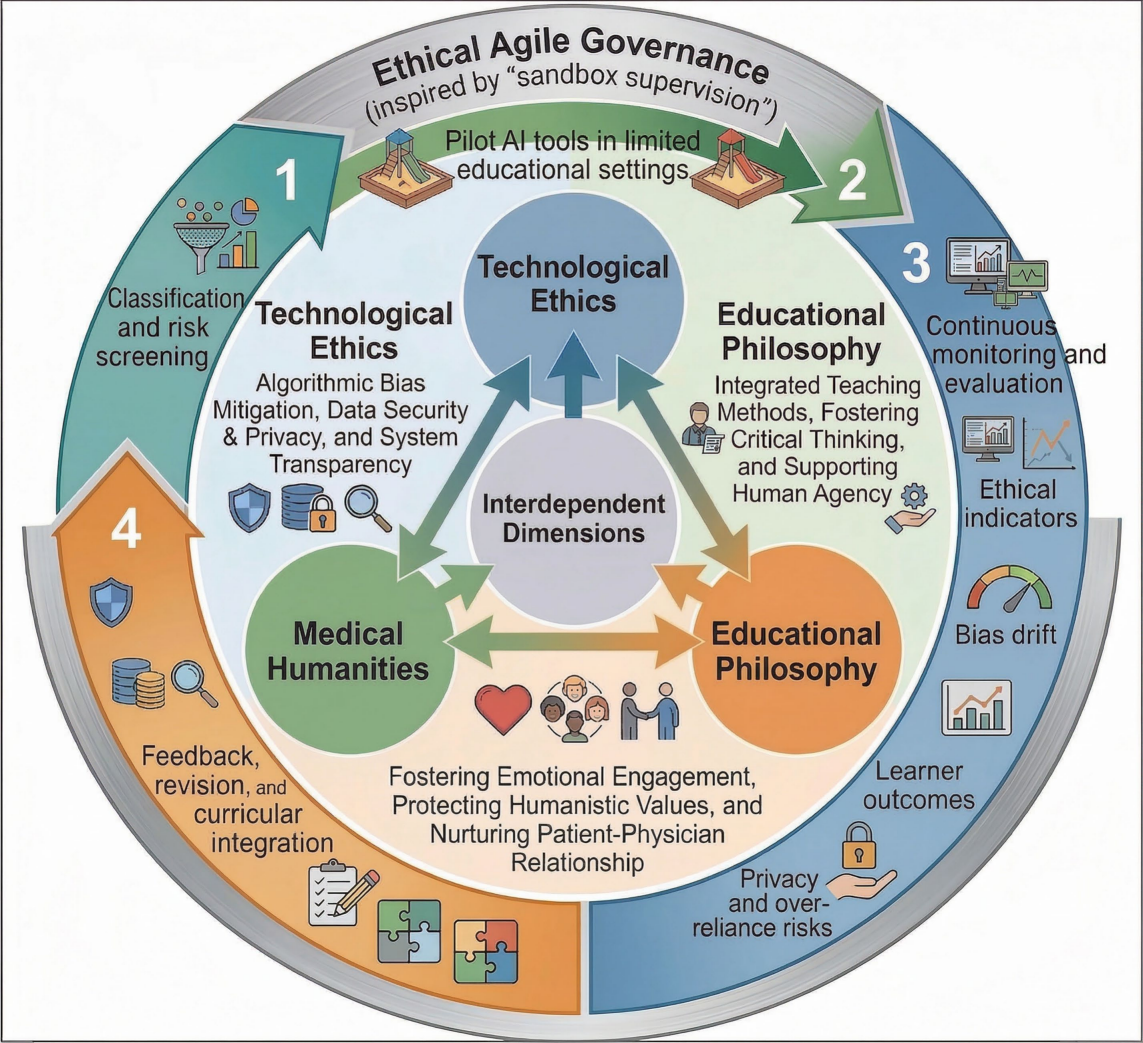
Three-Dimensional Ethical Governance Framework for AI in Medical Education. This figure illustrates the proposed governance framework for the responsible integration of AI into medical education. The framework consists of three interdependent dimensions: technological ethics, educational philosophy, and medical humanities. It also includes a dynamic Ethical Agile Governance mechanism inspired by “sandbox supervision,” through which AI tools undergo classification and risk screening, pilot use in limited educational settings, continuous monitoring and evaluation, and feedback-driven revision and curricular integration. Key monitoring domains include ethical indicators, bias drift, learner outcomes, and privacy and over-reliance risks. (Free for commercial use, as indicated on ShareIcon.net. Source: https://www.shareicon.net/).

To address the multifaceted ethical and practical challenges of AI integration in medical education, this paper proposes a three-dimensional ethical governance framework integrating technological ethics, educational philosophy, and medical humanities—a holistic model intended to move beyond fragmented approaches toward a more coordinated and systemic governance structure (Figure 2). This framework is designed to be dynamic and adaptive, in line with the rapid evolution of AI technologies and the changing needs of medical education (35, 36). The three dimensions are interdependent, and no single dimension is sufficient to ensure responsible AI integration in isolation.

### 5.1 Technological ethics dimension: technical standards and regulatory compliance

This dimension provides the foundation of the governance framework by focusing on the technical and regulatory standards applicable to AI tools used in medical education (4, 35). Key objectives include: (1) the clear classification of AI tools (e.g., generative AI vs. clinically validated systems), together with distinct guidance for their educational use (4, 5); (2) rigorous bias testing and validation of AI models, including transparent reporting of performance across diverse patient subgroups (10, 13); (3) compliance with data security and privacy regulations (e.g., HIPAA and GDPR), alongside the use of privacy-preserving approaches such as federated learning and homomorphic encryption where appropriate (8, 13); (4) the establishment of multidisciplinary oversight mechanisms involving medical educators, AI engineers, and bioethicists to evaluate AI tools before their adoption in medical education (4, 36).

### 5.2 Educational philosophy dimension: curricular integration and pedagogical alignment

This dimension focuses on aligning AI integration with the core educational philosophy of medical education: the training of competent and compassionate physicians (1, 19). Key objectives include: (1) incorporating structured AI literacy into medical curricula, including AI classification, performance evaluation, bias detection, and ethical use (13, 37); (2) integrating AI tools into traditional teaching methods rather than replacing them, so that AI-supported standardization is balanced with context-dependent clinical reasoning and human interaction (27, 29); (3) providing ongoing professional development for medical educators on the effective and critical use of AI in teaching (31, 37); (4) developing assessment strategies that evaluate students’ ability to use AI responsibly, for example by combining AI-assisted diagnostic performance with communication and humanistic care measures (32).

### 5.3 Medical humanities dimension: humanistic values and patient-centered care

This dimension represents the normative core of the framework and seeks to ensure that AI integration remains aligned with the fundamental humanistic values of medicine (12, 19). Key objectives include: (1) explicitly linking AI technical literacy to humanistic care in the curriculum, for example by teaching AI bias evaluation as a means of promoting patient equity (12); (2) using AI tools to support, rather than replace, humanistic care training, such as AI-assisted virtual patient simulations for communication practice (27); (3) establishing ethical guidance for AI use that prioritizes patient autonomy, safety, and appropriate human oversight in clinical decision-making (4); (4) fostering a culture of critical AI use among students and educators, in which AI is viewed as a supportive tool rather than a substitute for professional judgment (29). One possible discussion-based format is an AI and Humanism roundtable in which students and educators examine real or simulated cases of AI use in clinical practice and reflect on their humanistic implications (32).

### 5.4 Operationalizing the three-dimensional ethical governance framework in medical education

To translate the proposed framework into practice, medical education institutions should establish clear governance structures, phased implementation pathways, and measurable evaluation criteria.

A staged implementation pathway may further support the safe and effective integration of AI into medical education. First, AI tools intended for educational use should be classified according to their functions, validation status, and level of risk, such as generative AI, predictive algorithms, and clinically validated decision-support systems (7, 22). Second, selected tools may be piloted in limited educational settings, such as individual courses, clerkships, or simulation modules, under faculty supervision, with attention to indicators including accuracy, bias, student reliance, and communication performance (36, 38). Third, tools that demonstrate acceptable educational value and ethical safety may be incorporated into formal curricula, accompanied by faculty development, student assessment strategies, and data governance protocols (31, 37). Finally, continuous monitoring and periodic revision remain necessary to assess bias drift, privacy risks, student over-reliance, and possible effects on humanistic competencies over time (10, 38).

To support implementation, the framework should also be linked to measurable indicators across its three dimensions. Relevant indicators may include subgroup performance gaps, external validation status, and data security compliance under the technological ethics dimension; AI literacy assessment outcomes, case-based reasoning performance, and faculty training completion under the educational philosophy dimension; and empathy-related measures, communication OSCE performance, reflective evaluation of AI use, and patient-centered reasoning under the medical humanities dimension (10, 13, 32). A practical curricular pathway may involve introducing foundational AI literacy modules in the preclinical stage, AI-assisted diagnostic critique exercises during clinical training, and structured discussion of bias, patient autonomy, and responsibility in medical humanities teaching, with assessment combining OSCEs, reflective writing, and appraisal of AI-assisted clinical reasoning (27, 29, 32, 39).

Taken together, these operational elements help move the framework from a conceptual model toward an implementable governance approach. Supported by an adaptive mechanism inspired by “sandbox supervision” (38), the framework may provide medical education institutions with a flexible structure for evaluating emerging AI tools while maintaining continuous attention to ethical, educational, and humanistic outcomes.

## 6 Discussion

Historically, medical education has relied on the apprenticeship model, in which students learn through hands-on experience under the guidance of experts (1). However, as AI reshapes educational paradigms, medical education is gradually evolving from an experience-based apprenticeship model toward a more data-driven and technologically supported model (9). For example, virtual simulation training with highly precise anatomical modeling may enable medical students to practice hemostatic procedures for variceal bleeding in cirrhosis within advanced simulation environments (30, 40). Such physics-engine-based training scenarios may allow students to rehearse complex procedures that would traditionally require extensive clinical exposure, thereby improving learning efficiency and potentially reducing the risk of error in real clinical settings (30).

While AI has considerable potential in medical education, it also introduces significant ethical challenges, particularly with regard to privacy, bias, and the preservation of humanistic care (9, 19). At the technical level, the combined use of federated learning and homomorphic encryption offers an important privacy-protection paradigm (20). For example, distributed training models may allow hospitals to collaboratively develop AI systems for gastrointestinal bleeding detection without sharing original patient data, thereby helping to balance data utility with privacy protection in AI model development.

Looking ahead, AI in medical education may increasingly move toward more embodied and interactive forms of integration (27). A new generation of mixed reality (MR) training systems may further enhance surgical education by enabling stereoscopic visualization and annotation of robotic-assisted surgery procedures in immersive learning environments (27). Such systems allow teachers to record surgical procedures and share annotated videos with students, who can then view and interact with them through web-based platforms, personal mobile devices, or desktop stereo systems. The integration of virtual annotations and shared pointers can support real-time interaction between teachers and students, thereby enriching the learning experience. These systems may not only facilitate the acquisition of technical skills but also promote a deeper understanding of procedural workflows, supporting AI’s transition from a knowledge-assistance tool to a more interactive educational collaborator in medical education (27).

At the same time, the growing use of generative AI in tasks such as drafting clinical progress notes for gastrointestinal bleeding cases raises new legal and ethical questions, including responsibility for AI-generated content and the ethical status of virtual patients (6). For this reason, a dynamic mechanism for the evolution of ethical norms remains important (36, 38). An “Ethical Agile Governance” framework, inspired by the “sandbox supervision” model developed by the Singapore AI Ethics Institute, may provide a practical pathway in which new technologies are explored in limited teaching scenarios while real-time ethical indicators, such as students’ empathy changes and algorithmic bias drift rates, are monitored (38). Within such an approach, AI should not be treated merely as a tool for efficiency, but as a technology whose educational use remains aligned with humanistic values in medical practice (8). Accordingly, AI models should be subject to appropriate ethical evaluation to reduce the risk of bias and misinformation affecting medical professionals and students (10).

Moreover, the application of AI in medical education requires continuous evaluation and adaptation (9). Educators need the knowledge and tools to critically evaluate and incorporate AI into their teaching practices, while students should be trained to use AI responsibly (29, 31). By fostering critical thinking and encouraging medical students to value the uniquely human aspects of healthcare, such as empathy and communication, medical education can better ensure that AI enhances rather than displaces these essential capacities (29).

## 7 Conclusion

The integration of AI into medical education offers substantial opportunities to enhance training and improve patient care, but it also raises important ethical and practical challenges that require careful and proactive management. A key challenge in current AI integration is the insufficient distinction between generative AI tools and clinically validated AI systems, together with the lack of concrete training in AI-related competencies, such as evaluating model performance and recognizing algorithmic bias (4, 5, 10, 13). Technological competence and humanistic care should not be treated as opposing domains; rather, AI literacy forms an important component of contemporary humanistic medical practice by helping to protect patients from AI-related risks (12, 41).

The proposed three-dimensional ethical governance framework, integrating technological ethics, educational philosophy, and medical humanities, together with a dynamic Ethical Agile Governance approach, may support a more balanced integration of AI in medical education (35, 38). At the same time, further work is needed to operationalize and evaluate this framework in specific curricular contexts and across diverse institutional settings. Its implementation will require coordinated efforts from multiple stakeholders, including educators, students, developers, and policymakers, to support responsible AI use, appropriate oversight, and context-sensitive educational design (4).

Ultimately, the goal of integrating AI into medical education is not to replace human practitioners with machines, but to support the development of physicians who are both technically competent and grounded in humanistic values (19, 29, 36). With appropriate governance, curricular refinement, and sustained attention to ethical and humanistic principles, AI may complement traditional medical education and contribute to the training of competent, compassionate, and AI-literate healthcare professionals.
